# Simulating Multi-Scale Pulmonary Vascular Function by Coupling Computational Fluid Dynamics With an Anatomic Network Model

**DOI:** 10.3389/fnetp.2022.867551

**Published:** 2022-04-25

**Authors:** Behdad Shaarbaf Ebrahimi, Haribalan Kumar, Merryn H. Tawhai, Kelly S. Burrowes, Eric A. Hoffman, Alys R. Clark

**Affiliations:** ^1^ Auckland Bioengineering Institute, University of Auckland, Auckland, New Zealand; ^2^ Department of Radiology, University of Iowa, Iowa City, IA, United States

**Keywords:** pulmonary circulation, computational fluid mechanics, network flow modelling, lung, computational model

## Abstract

The function of the pulmonary circulation is truly multi-scale, with blood transported through vessels from centimeter to micron scale. There are scale-dependent mechanisms that govern the flow in the pulmonary vascular system. However, very few computational models of pulmonary hemodynamics capture the physics of pulmonary perfusion across the spatial scales of functional importance in the lung. Here we present a multi-scale model that incorporates the 3-dimensional (3D) complexities of pulmonary blood flow in the major vessels, coupled to an anatomically-based vascular network model incorporating the multiple contributing factors to capillary perfusion, including gravity. Using the model we demonstrate how we can predict the impact of vascular remodeling and occlusion on both macro-scale functional drivers (flow distribution between lungs, and wall shear stress) and micro-scale contributors to gas exchange. The model predicts interactions between 3D and 1D models that lead to a redistribution of blood between postures, both on a macro- and a micro-scale. This allows us to estimate the effect of posture on left and right pulmonary artery wall shear stress, with predictions varying by 0.75–1.35 dyne/cm^2^ between postures.

## Introduction

The pulmonary circulation carries almost the entire cardiac output to the pulmonary alveoli, in order to expose deoxygenated blood to the higher partial pressure of oxygen in the alveolar airspaces. Its function is truly multi-scale, in that blood traverses through vessels of 2–3 cm diameter at the main pulmonary artery Edwards et al. (1998) down to the order of *μ*m diameters in the pulmonary capillaries Fung and Sobin (1969). The distribution of blood flow within this circulation is critical to providing good matching of perfusion to ventilation (air flow) at the alveolar level. This matching is determined by a combination of the effect of gravity acting to deform lung tissue locally, the hydrostatic effect of gravity which acts directly on blood, and a contribution from the anatomic structure of the pulmonary airways and blood vessels Kang et al. (2018). The distribution of perfusion has been demonstrated to be dependent on each of these mechanisms Clark et al. (2011b); Hlastala and Glenny (1999); Hopkins et al. (2007); West et al. (1964), with anatomic structure playing a greater role in the distribution of perfusion than ventilation in the normally functioning adult lung Clark et al. (2011b); Kang et al. (2018). Overall, the nature of blood flow in the largest and smallest blood vessels is different, and this means that the physics of computational models derived to capture their function relies on different assumptions and biomechanical models. For example, blood flow in the main pulmonary arteries has significant 3-dimensional (3D) complexities, and is functionally altered in patients with pathologies such as pulmonary hypertension Schäfer et al. (2017). In contrast, flow rates and Reynolds numbers are lower in the pulmonary micro-circulation, leading to reduced complexity in flow patterns. However, the pulmonary capillaries directly interact with the expanding alveoli, and are recruited and de-recruited dynamically in response to changes in local air and blood pressure Fung and Sobin (1969); West et al. (1964); Yen et al. (1980).

Several computational models of the branching network of arteries and veins in the pulmonary circulation have been proposed. These range from 3D computational fluid dynamics (CFD) simulations representing the branching network of large arteries Kheyfets et al. (2015); Tang et al. (2011, 2012), through to 1-dimensional (1D) network models that aim to capture the distribution of blood flow within the entire pulmonary circulation Clark et al. (2011b, 2014); Ebrahimi et al. (2021). These models typically target two main areas: 1) wall shear stress (WSS) distribution in pulmonary artery networks which is intimately associated (and correlated) with endothelial dysfunction Kheyfets et al. (2015); Tang et al. (2012), but that cannot be measured experimentally; 2) the major drivers of pulmonary perfusion distribution in the lung in health and disease, and how local perfusion contributes to ventilation-perfusion matching and gas exchange Clark et al. (2014); Kang et al. (2018). 3D CFD is far more accurate in predicting WSS than simplified 1D models. One of the well known challenges of such models is the boundary condition prescription, with small deviations in boundary conditions sometimes yielding large differences in observed velocities Kheyfets et al. (2013). 1D networks on the other hand can well-predict micro-scale perfusion and the impact of vascular structure and gravity on this function, but cannot be used to accurately simulate flow in the major pulmonary arteries Clark et al. (2017). In pathological lungs, this is particularly important, as micro-vascular changes may impact pressure, flow and WSS in the major vessels, and vice versa Wang and Chesler (2011). Previous models have coupled 3D CFD to downstream models via imposed boundary conditions, for example by a structured tree approach Kheyfets et al. (2015), or in other organ systems by using measured values of flow over time from Doppler ultrasound as boundary conditions Oshima et al. (2001); Perktold and Rappitsch (1995). However, no model exists that can predict the multi-scale function in the complex network of the pulmonary vasculature across spatial scales.

Here, we present an integrated model of the pulmonary circulation that includes a 3D representation of the major pulmonary arteries coupled to an anatomically realistic 1D network model that comprises the entire circulation that lies downstream of these major arteries. Importantly, the network model incorporates each of the major contributors to perfusion distribution in the lung (anatomical structure across scales, and gravitational effects), alongside the capability to predict WSS accurately in the major pulmonary arteries.

## Methods

**FIGURE 1:**
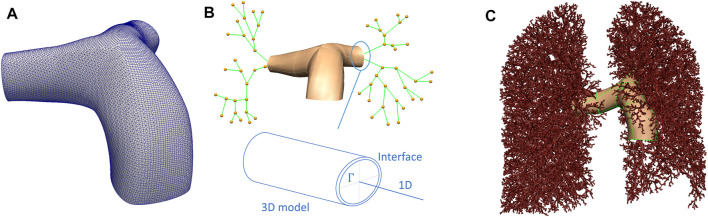
Schematic representation of the 3D/1D model of the pulmonary circulation. **(A)** The 3D model mesh. **(B)** A schematic of how 3D and 1D models are related, including an illustration of the interface, Γ, between the two models. **(C)** Depicts the whole lung model, with the largest blood vessels explicitly meshed in 3D, and the smaller blood vessels represented by elements defined by branch points and their radii.

The methodological framework proposed here employs a subject-based model that represents the anatomical structure of an individual’s lungs generated from computed tomography (CT) imaging. An illustration of the 3D geometrical model and its connectivity to a 1D network model is shown in Figure 1. The methodology is demonstrated in a whole lung with the main pulmonary artery and left and right pulmonary arteries simulated as 3D structures. While the number of generations that may be incorporated into the 3D model is arbitrary, two generations of arteries were chosen in this study to test the hypothesis that posture may impact the distribution and nature of flow between the left and right lungs. The 3D structure is connected to an anatomically-based 1D tree representing morphological branching to the level of the pulmonary acini. For this study, the model geometry was derived from CT images of a healthy adult male (age: 23; weight: 80.9 kg; body mass index: 23.1 kg/m^2^) representative of a population of 30 normal subjects aged between 20–30 years old, derived from the Human Lung Atlas Database Hoffman et al. (2004). The subject has the closest lung shape to the mean lung shape for this population determined by a principal component analysis Osanlouy et al. (2020). Functional Residual Capacity (FRC) measured seated was 3.4 L. Imaging was acquired supine with lung volume held constant at 50% of vital capacity.

### Model Geometry

The model geometry employed in this study aims to capture the lung shape and the distribution of the largest blood vessels to one generation beyond the sub-segmental level, as measured from CT imaging. The first two generations of blood vessels were represented by their 3D structure, then the centerlines of blood vessels were derived from CT to one generation beyond the segmental level. Blood vessels beyond this level, to the level of the pulmonary acinus are generated as a branching network that is consistent with morphometric data on typical branching structures of pulmonary arteries and veins Burrowes et al. (2005); Horsfield (1978); Horsfield and Gordon (1981); Huang et al. (1996); Tawhai et al. (2004). Lungs, lobes, airways and intra-pulmonary blood vessels were segmented using PASS (Pulmonary Analysis Software Suite, University of Iowa). This study used non-contrast-enhanced imaging, therefore extra-pulmonary blood vessels were segmented manually, starting from the point of attachment of the main pulmonary artery to the heart. Three scales of model geometry were created. The first represents a 3D volume mesh of the first two generations of pulmonary arteries. The second is a spatially distributed network of 1D elements that represents segments of the centerlines of the branching vascular tree. The final scale represents the acinar structure.

Figure 1 provides a schematic of the interface between the 3D volume and the 1D representation of the blood vessel network. A 3D surface model is generated to reflect the vessel surface of the main pulmonary artery and its left and right branches (Figure 1A). A centerline representation of the main pulmonary artery and distal branches to one generation beyond the segmental level was extracted, and a 1D network template mesh was manipulated to assign nodal locations to each bifurcation of the pulmonary arteries down to one level beyond the segmental arteries (Figure 1B). The surface of the 3D geometry has a bicubic-Hermite element structure, and this is fit to a cloud of datapoints representing the vessel surface by a least squares minimization process - optimizing the sum of squared differences between each surface point and the nearest surface element Fernandez et al. (2004); Tawhai et al. (2009). The surface was converted to a volume mesh using CFMesh (version 1.1, Creative Fields, United Kingdom)—a library implemented in OpenFOAM for mesh generation (Version 7, OpenCFD Ltd.^1^). The meshing process produces hexahedral cells, with polyhedra in the transition zones between cells of various sizes with hexahedral elements at the boundary layers.

To generate a morphological vascular network beyond the major vessels, the volume filling branching algorithm proposed by Tawhai et al. (2004), and presented for the pulmonary blood vessels by Burrowes et al. (2005) was employed. The segmentations of the lobes were converted to a surface data cloud, and a bicubic-Hermite template surface mesh was fitted (following Tawhai et al. (2009)). The surface mesh was filled with an equi-distributed array of datapoints. Using the branching upper vasculature derived from imaging (to one generation beyond the segmental level) as an initial condition, a volume filling algorithm was used to generate branching vessels that fill the volume, and that terminate at ≈ 32,000 terminal blood vessels feeding the pulmonary acini. For simplicity, the pulmonary venous structure is assumed to follow the pulmonary arterial structure except at the pre-segmental level (which is derived manually from imaging). Each blood vessel represented in the 1D network model is described by an element representing its centerline, and its radius.

The acinus is modeled as a 9-generation symmetric network of arterioles and venules that are connected in series, and are connected in parallel by ‘sheets’ of capillary bed (Clark et al. (2010); Fung and Sobin (1969); Haefeli-Bleuer and Weibel (1988)). This anatomically-based intra-acinar structure has been termed a ‘ladder’ model (Clark et al. (2010; 2011a)). A symmetric structure is assumed to allow the model solution in ≈30,000 acinar units, and our previous modeling suggests that while within-acinus branching asymmetry impacts sub-acinar heterogeneity in perfusion, its impact on acinar resistance is small Clark et al. (2011a). It allows a direct connection between the capillary structure (which is influenced primarily by local air pressure and inflation), and a physiological stratification of function within them from the most proximal to distal capillary (Read (1969a,b)). The ladder model also facilitates coupling micro-circulatory function to the intra-pulmonary macro-vasculature (which is under the influence of lung tethering pressure, related to elastic recoil) and outwards to the extra-pulmonary vessels with their more complex flow patterns and direct connection to the heart.

### 3D Computational Fluid Dynamics Simulations

To simulate flow in the pulmonary arteries, the 3D CFD solver OpenFOAM was used. The blood within these arteries was assumed Newtonian, incompressible and laminar. The PimpleFoam solver based on the PIMPLE algorithm was used Passalacqua and Fox (2011). This algorithm combines the PISO (Pressure-Implicit Splitting Operator) Issa (1986) and SIMPLE (Semi-Implicit Method for Pressure-Linked Equations) algorithms Caretto et al. (1973); Ferziger et al. (2002); Jasak (1996); Passalacqua and Fox (2011); Patakar (1980). Outer correction loops are used in the PIMPLE method to specify number of iterations. To guarantee that the explicit sections of the equations converge, outer corrector loops are enabled. In the PIMPLE algorithm, a dynamic time step technique, allows the time step to vary in relation to the maximum Courant number allowed. Courant number is a dimensionless measure that provides the rate at which data is transported from one cell to another. Adjustable time step is utilized in all of the current simulations, with a maximum Courant number of 1.0. Steady state simulations were performed. Flow inlet boundary conditions were prescribed at the main pulmonary artery (the inlet), and fixed pressure boundary conditions were imposed at each outlet of the 3D model, when not coupled to the 1D model. A mesh dependence analysis on the 3D model was performed to ensure that the mesh resolution does not affect the final results.

To estimate WSS on the pulmonary vasculature, a quantitative metric was chosen that is considered to be independent of the 3D model reconstruction Kheyfets et al. (2015). The WSS magnitude is averaged over the luminal surface (*S*) of the 3D geometry to define a spatially averaged wall shear stress (SAWSS)

$$\text{SAWSS}=\frac{1}{A}\iint_{S}\text{WSS }\partial S,$$ (1)

where *A* is the total surface area of the luminal surface of the mesh.

### 1D Network Flow Simulations

The 1D network flow model, that allows for a functional connection between the macro- and micro-vasculatures is based on a compliant electrical analogue described in detail by Clark et al. (2011b) This model is available as an installable library within the lungsim library of Aether.^2^ The model incorporates key features of extra-capillary anatomy and blood flow, and employs a sheet flow model for the pulmonary capillaries (derived by Fung Fung and Sobin (1969)) which includes the recruitment and derecruitment of capillary bed in response to air and blood pressures. In each extra-capillary blood vessel the relationship between blood pressure and blood flow $\left(\dot{Q}\right)$ is described by a modified Poiseuille equation that accounts for the impact of gravity on blood flow

$$\Delta P=\frac{128\mu L}{\pi D^{4}}\dot{Q}+\rho_{b}gL\cos\theta ,$$ (2)

where Δ*P* is the blood pressure drop along the length of the vessel, *μ* is the viscosity of blood, *L* is the axial length of the vessel, *D* is the diameter of the vessel, *ρ*_*b*_ is the density of blood, *g* is gravitational acceleration, and *θ* is the angle the vessel centerline makes with the direction of gravity. The gravitational term is neglected in small intra-acinar vessels where the resistance term dominates. A linear relationship between transmural pressure (*P*_*tm*_), defined as blood minus extra-vascular pressure, is assumed with compliance constant *α* Clark et al. (2011b); Krenz and Dawson (2003). In vessels with *D* < 200 *μ*m the extra-vascular pressure is defined as alveolar pressure Yen et al. (1980), and in larger vessels, extra vascular pressure is defined as local elastic recoil, which is assumed in this model to vary linearly with gravitational height.

At the level of the capillary sheet, flow depends on the local balance between blood and air pressures, consistent with West’s West et al. (1964); West (1999) description of zones of capillary flow in the lungs. With *P*_*tm*_ at this scale defined as blood minus air pressure, we are able to define a capillary sheet height *H* across a range of capillary recruitment conditions

$$H=\left\{\begin{array}{cc} 0,\; & \text{if}\;P_{tm}<0,\\ H_{0}\left(1+\alpha_{c}P_{tm}\right)\; & \text{0}\leq P_{tm}<P_{CU},\\ H_{max}=H_{0}+\alpha_{c}P_{tm},\; & \text{if}\;P_{CU}\leq P_{tm}, \end{array}\right.$$ (3)

where *P*_*CU*_ is defined as an upper bound for pressure beyond which the sheet height remains constant. The relationship between pressure and flow is then defined by

$$\dot{Q}=\frac{SA}{\mu_{c}fl_{c}^{2}}\int H^{3}dP_{tm},$$ (4)

where *SA* is the capillary surface area in any given sheet of capillaries connecting arteriolar to venular circulations, *μ*_*c*_ is the viscosity of capillary blood, *f* is a constant, *l*_*c*_ is the average path length between arteriole and venule within the capillary sheet. Analytical relationships between $\dot{Q}$ and *P* are derived in detail by Clark et al. (2010) for conditions relevant to pulmonary capillary perfusion. At each bifurcation in the 1D network, continuity of pressure and conservation of flow (flow into a bifurcation equals flow out) is prescribed. In the absence of coupling to the 3D model, cardiac output is specified as a flow boundary condition at the main pulmonary artery, and a pressure outlet condition is imposed at the main pulmonary veins.

### Coupling Method

There are two interfaces connecting the two domains at the outlets of the left and right pulmonary arteries in this model (Figure 1), however, any number of interfaces can be defined. At each interface Γ a circle of Willis methodology is employed, following Passerini et al. (2009). Coupling was achieved by prescribing continuity of pressure and flow defined by

$$P_{1D}=\frac{1}{\left|\Gamma \right|}\int_{\Gamma }p_{3D}d\gamma ,\;\;\;{\dot{Q}}_{1D}=-\rho \int_{\Gamma }u_{3D}.nd\gamma ,$$ (5)

where indexes 1*D* and 3*D* represent corresponding domains (Figure 1B). *γ* represents an interface surface segment and *u* and *n* are velocity and surface normal vectors.

**FIGURE 2:**
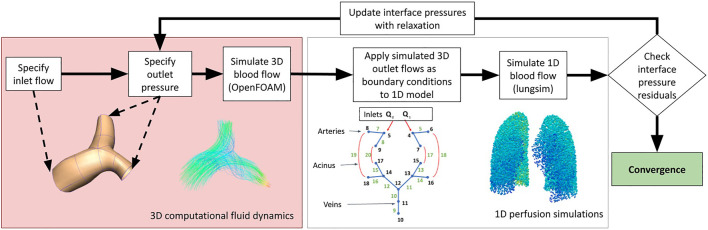
A schematic illustrating the methodologies employed in this study to couple 3D computational fluid dynamic simulations to 1D perfusion in a network model incorporating the multiple scales of function that are important to blood flow in the lungs.

To solve the coupled system, we solve the two systems iteratively. There is a matching interface in the geometry at the point of intersection between models, and simulations in the 1D model are conducted with boundary conditions imposed at these interface points. The 3D model is simulated by prescribing a boundary condition $\left({\dot{Q}}_{inlet}\right)$ and pressure-outlet boundary conditions (*P*_*i*_, where *i* is the number of outlets in the 3D geometry, or interfaces). Solution of 3D governing equations yields corresponding outlet flows $\left({\dot{Q}}_{i}\right)$ . These flows are passed to the 1D network as inlet flow boundary conditions, and the 1D network solution is obtained. The 1D solution yields corrected pressure values $\left(P_{i}^{1}\right)$ that are passed to the 3D model as outlet boundary conditions for the second coupling iteration, and so on. Additionally, at each coupling iteration, the corrected pressure and flow are regularized using an under-relaxation factor

$$\left\{\begin{array}{c} {P_{i1D}}^{k+1}=\lambda {P_{i3D}}^{k}+\left(1-\lambda \right){P_{i1D}}^{k},\text{and,}\;\\ {Q_{i1D}}^{k+1}=\lambda {Q_{i3D}}^{k}+\left(1-\lambda \right){Q_{i1D}}^{k},\; \end{array}\right.$$ (6)

where *λ* is the under-relaxation factor, *i* is the interface index, and *k* is the coupling iteration number between domains. The solution procedure is illustrated in Figure 2.

### Coupling Convergence

Convergence is assumed to have been reached when the pressure and flow at the interface branches are within a specified tolerance between two coupling iterations. Continuity at the 1D-3D domain interface between iterations was used to define error. If we define *ϵ*, which is a user-defined tolerance, to be the error threshold for convergence, the solution is converged when

$$\sqrt{\underset{i}{\sum }{\left(P_{i}^{k}-P_{i}^{k-1}\right)}^{2}}\leq \epsilon ,$$ (7)

is satisfied, with $P_{i}^{k}$ being the pressure at interface *i* after the *k*th iteration. Here, *ϵ* is chosen to be 0.1 Pa which is the cumulative error allowed at both interfaces. This threshold ensures that the error is computationally acceptable compared with typical pressure values at this scale (≈2000 Pa in healthy, and higher in hypertensive scenarios).

### Model Parameterization and Simulations Conducted

We conduct simulations to assess the behavior of the model under two primary perturbations: 1) the effect of cardiac output within a physiological range, and 2) the effect of posture. Simulations were conducted with boundary conditions at the main pulmonary artery reflecting a mean volumetric flow of 4, 4.8 and 5.6 L/min, in each of zero gravity, prone, supine and upright postures. Primary output metrics were the distribution of acinar perfusion, and SAWSS in the major pulmonary vessels (the main pulmonary artery and the left and right pulmonary arteries). Model parameters and key geometrical features of the model are given in Table 1.

**TABLE 1:** A description of model parameters, and geometric features of the 3D model. The parameterization of the 1D network model is described in detail by Clark et al. (2011b), with key parameters outlined here.

Parameter	Description	Value	References & Methodology
*Α*	1D extra-acinar compliance (Pa^−1^)	1.49 × 10^−4^	Krenz and Dawson (2003): Derived from 26 studies of distensibility across species
*ρ*_*b*_	Blood density (kg/m^3^)	1050	Pries et al. (1996): Population average across *in vitro* studies
*Μ*	Blood viscosity (Pa/s)	0.0035	Pries et al. (1996): Population average across *in vitro* studies
*μ*_*c*_	Capillary blood viscosity (Pa/s)	0.0019	Fung (1984): Combination of *in vitro* measurements and theoretical derivation
*G*	Gravitational acceleration (m/s^2^)	9.81	
*SA*	Total capillary surface area (m^2^)	65.4	Gehr et al. (1978): Population average derived from electron microscopy
*α*_*c*_	Capillary sheet compliance (m/Pa)	1.3 × 10^−9^	Sobin et al. (1979): Estimate from photomicrographs in *ex vivo* cat lung
*F*	Numerical factor (no units)	21.6	Fung and Sobin (1972): Theoretical estimation
*l*_*c*_	Average pathlength from arteriole to venule (m)	1186 × 10^−6^	Zhou et al. (2002): Theoretical estimation
*H*_0_	Unstrained capillary sheet height (m)	3.5 × 10^−6^	Sobin et al. (1979): Estimate from photomicrographs in *ex vivo* cat lung
*H*_max_	Maximum capillary sheet height (m)	7.7 × 10^−6^	Sobin et al. (1979): Estimate from photomicrographs in *ex vivo* cat lung
	Strahler diameter ratio (arteries)	1.52	Huang et al. (1996): Fit parameter to produce geometry consistent with vascular casting
	Strahler diameter ratio (veins)	1.56	Huang et al. (1996): Fit parameter to produce geometry consistent with vascular casting
	Main pulmonary artery area (m^2^)	7.72 × 10^−4^	Derived from CT.
	Left pulmonary artery area (m^2^)	3.77 × 10^−4^	Derived from CT.
	Right pulmonary	3.54 × 10^−4^	Derived from CT.
	3D geometry volume (m^3^) artery area (m^2^)	7.967 × 10^−5^	
	Number of 1D vessel elements	153396	Output from meshing
	Number of interfaces between 1D and 3D	2	User defined
	Number of acinar units	30676	Output from meshing, consistent cwith vascular casting Huang et al. (1996)

Results are presented first for the non-coupled *1D model* (as presented in Clark et al. (2011b)), and *3D model* (full CFD model in major arteries with fixed pressure boundary conditions) to understand non-linearity in the two systems. Then, the full model is presented as the *coupled model*, to analyze how whole lung perfusion simulations behave. The *zero gravity* (0g) coupled model can be interpreted as aligning with existing CFD strategies which assign resistance to outlets depending on the size of these vessels, or estimates for downstream resistances such as structured trees, as they represent the downstream resistance based on anatomy but in the absence of gravitational factors. Comparisons of CFD behaviors against data from the literature are provided in Supplementary Section S1.

## Results

### Mesh Independence

Table 2 shows mesh quality and changes in key output metrics (mean pulmonary artery pressure (mPAP), right pulmonary artery flow (RPA flow), left pulmonary artery flow (LPA flow) and SAWSS) for assessment of mesh independence of solutions. In total, six different mesh densities were generated to assess mesh independence of the model. For cell numbers > 199268 changes in each key output metric with further mesh refinement were < 2% and so this mesh density was selected for further simulations.

**TABLE 2:** Mesh quality and independence metrics. Quality metrics are: Number of faces on vessel wall, Maximum cell orthogonality for each mesh, average orthogonality of cells and maximum cell skewness. Key output metrics are: Mean pulmonary artery pressure (mPAP), right pulmonary artery flow (RPA flow), left pulmonary artery flow (LPA flow) and spatially averaged wall shear stress (SAWSS). Percent differences from the most refined mesh (340056 cells) are also reported.

Number of cells	26597	95081	151469	199268	270686	340056
Number wall faces	4178	12035	17421	21645	28151	34345
Average area of s single wall cell (mm^2^)	0.029	0.0081	0.0051	0.0039	0.0029	0.0023
Maximum orthogonality	64.49	64.87	64.27	64.69	64.62	64.49
Average orthogonality	9.31	7.32	6.60	6.38	6.21	6.19
Maximum skewness	2.85	3.06	2.83	2.72	2.69	2.70
mPAP (Pa)	2213.0	2211.5	2212.8	2211.8	2211.9	2211.9
mPAP (% difference from refined mesh)	0.05%	−0.02%	0.04%	−0.005%	0.001%	—
RPA flow (L/min)	2.629	2.641	2.711	2.645	2.646	2.645
RPA flow (% difference from refined mesh)	−0.60%	−0.15%	2.50%	−0.002%	0.04%	—
LPA flow (L/min)	2.171	2.159	2.089	2.155	2.154	2.155
LPA flow (% difference from refined mesh)	0.74%	0.19%	−3.06%	0.03%	−0.04%	—
SAWSS (dyne/cm^2^)	5.29	5.58	5.72	5.85	5.94	5.96
RPA flow (% difference from refined mesh)	−11.2%	−6.38%	−4.03%	−1.85%	−0.34%	-

### Flow-Pressure Relationships of 1D and 3D Model

**FIGURE 3:**
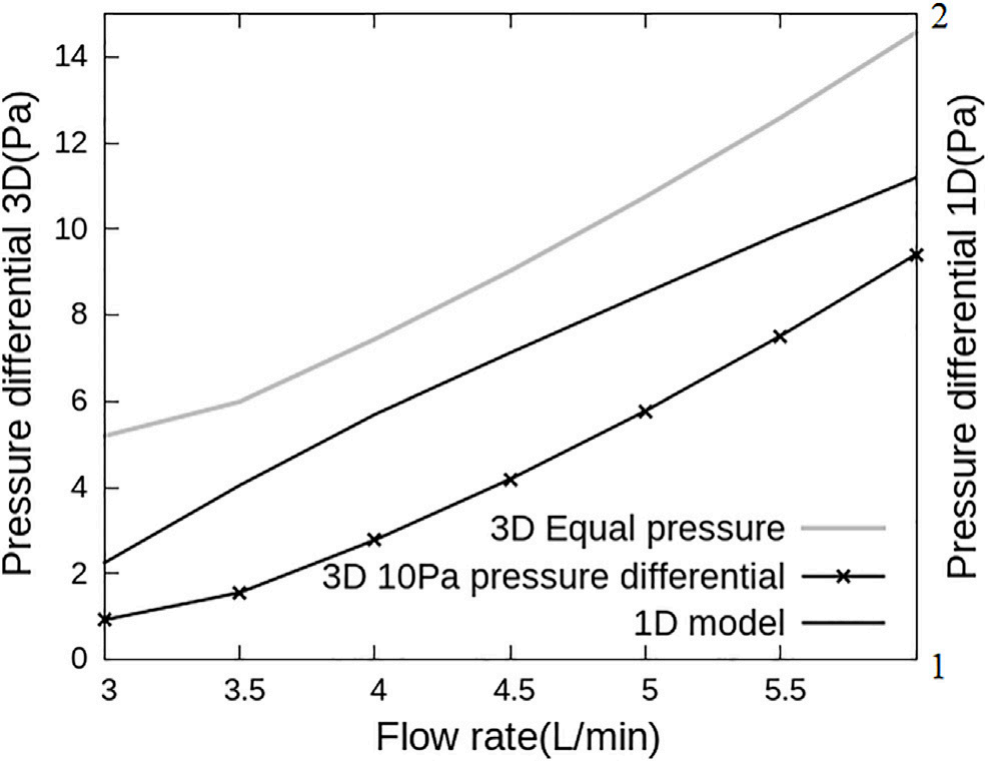
Flow and pressure relationships for the 1D and 3D models used in this work. Pressure differential is defined as the difference in pressure between inlet and LPA outlet. This differential is plotted versus inlet flow rate with 3D model simulations on the primary and 1D model simulations on the secondary axis. The pressure-flow association are different for the two models, with the 3D model dominated by a flow dependent resistance and the 1D model responding to resistance-compliance relationships.

The 1D and 3D models were first analysed independently to understand the effect of geometry on the flow-pressure relationship (Figure 3). The 1D model was solved under baseline parameterization and the pressure differential between the inlet and the outlets to the 3D model was reported, and the 3D model was solved first with fixed and equal pressure boundary conditions at the outlets, and again with a 10 Pa pressure differential between LPA and RPA to establish an asymmetry in the model. Both models show non-linear behavior, with the 3D model exhibiting relatively small changes in pressure with increases in flow at low flow rates than at high flow rates. The 1D model shows a flattening off of the relationship between flow and pressure at high flow rates.

When the 1D and 3D models are solved independently, each model also predicts a different distribution of blood flow between LPA and RPA. The 1D model is dominated by downstream effects due to vascular anatomy, capillary recruitment and gravity, and as such across all postures that were simulated, 46.2%–46.9% of flow is predicted to flow to the LPA. In the 3D model, 52.5%–53.2% of the flow is predicted to go through LPA under equal pressure boundary conditions and 28.2%–43% of the cardiac output flows through the LPA under a 10 Pa pressure difference between LPA and RPA outlets, depending on the cardiac output value. This is because the 3D model flow and pressure drop are affected primarily by geometrical detail such as curvature at the bifurcation. This difference between the 1D and 3D model predicted flows simply shows the importance of capturing subject-specific large scale effects into network models of pulmonary perfusion, and vice versa.

### Macro-Scale Blood Flow

**FIGURE 4:**
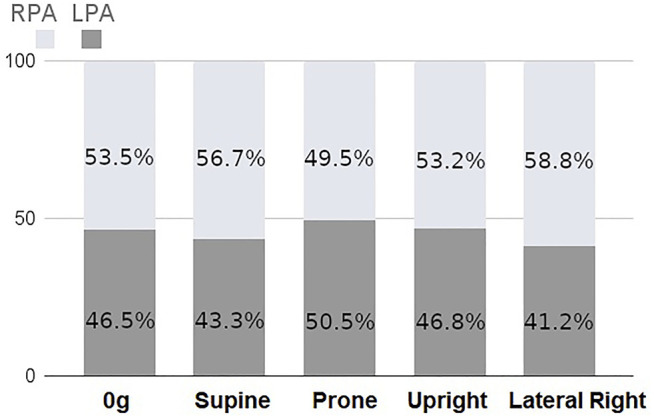
Percentage of total inlet blood flow (cardiac output) between left and right lungs as predicted by the coupled model with a fixed inlet flow of 4.8 L/min. There is a predicted redistribution of flow with posture due to gravitational effects.

The coupled model integrates macro-scale flow dynamics from the 3D model with perfusion in smaller blood vessels from the 1D model. This leads to a physiologically meaningful impact of gravity arising in the model across scales which is unique to this coupled system. Figure 4 shows the proportion of the cardiac output that enters the right and left lung in simulations of zero gravity (0g), and in 1g in prone, supine, upright and right-lateral, all with a fixed cardiac output of 4.8 L/min. For reference, in this model the right lung comprises 52.5% of the total lung volume. When model posture is altered there is a redistribution of blood, due to gravitational distribution of lung tissue with respect to the feeding vessels (that arise from the heart) and so the left and right lung flow balance is altered. This redistribution is to the left lung in prone, the right lung in supine and the left lung in left-lateral postures. Trends predicted by the model are consistent with data illustrating left-right flow distribution in the lungs derived from magnetic resonance imaging Wieslander et al. (2019). In zero gravity simulations (and in the 1D model presented by Clark et al. (2011b)) flow is distributed between left and right lungs proportionally to lung volume, with 53.5% of volumetric blood flow to the right lung.

**FIGURE 5:**
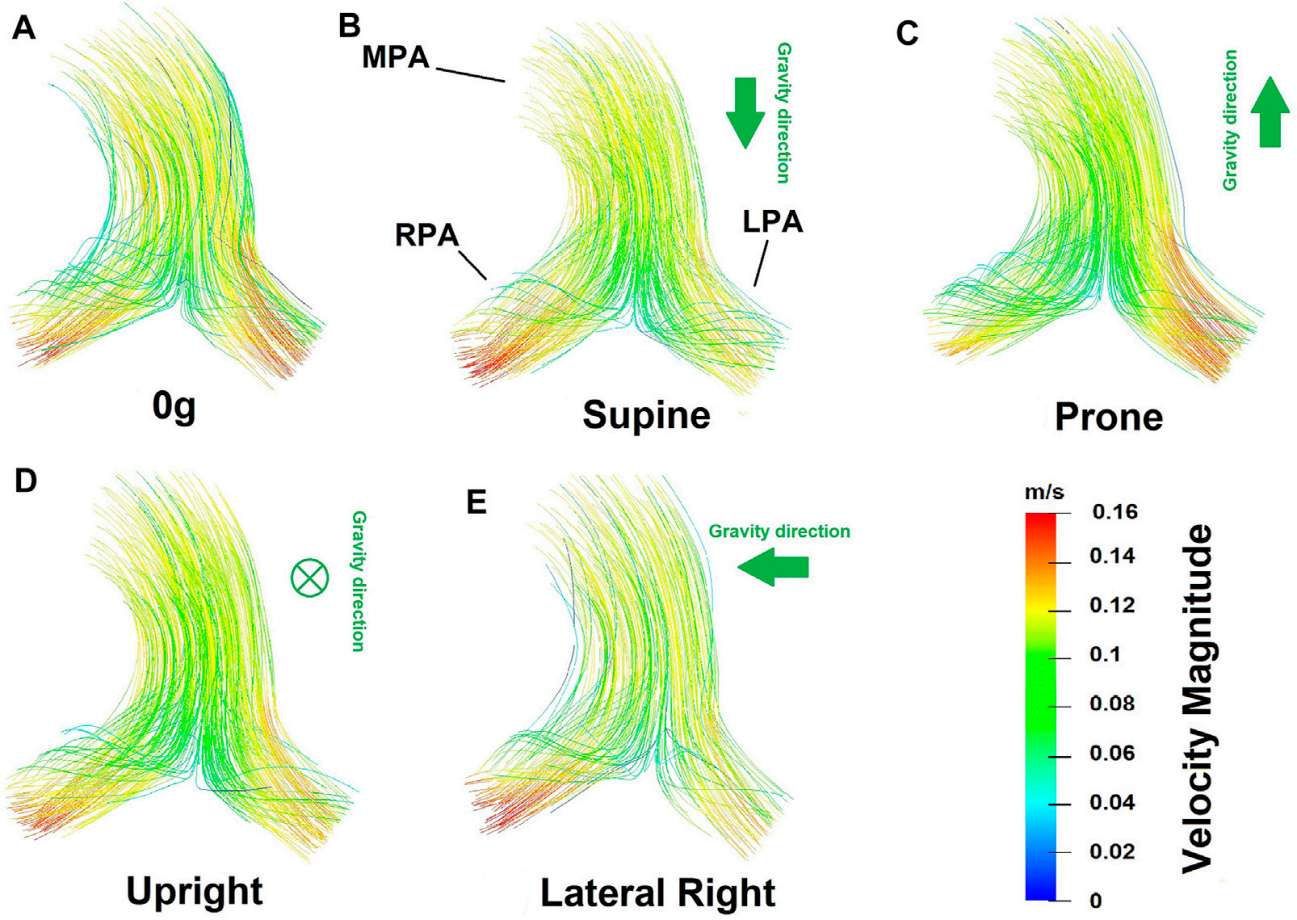
Flow velocity magnitude and streamlines predicted from the coupled model in different postures **(A)** zero gravity, **(B)** supine, **(C)** prone, **(D)** upright and **(E)** lateral right. In these images the viewer is looking down (into the page) at the main pulmonary artery. Therefore in supine simulations gravity is acting downwards with respect to the page, in prone simulations upwards, and in upright simulations into the page. The left-right shifts in blood flow distribution predicted in the model are evident in the flow velocity magnitude.

**FIGURE 6:**
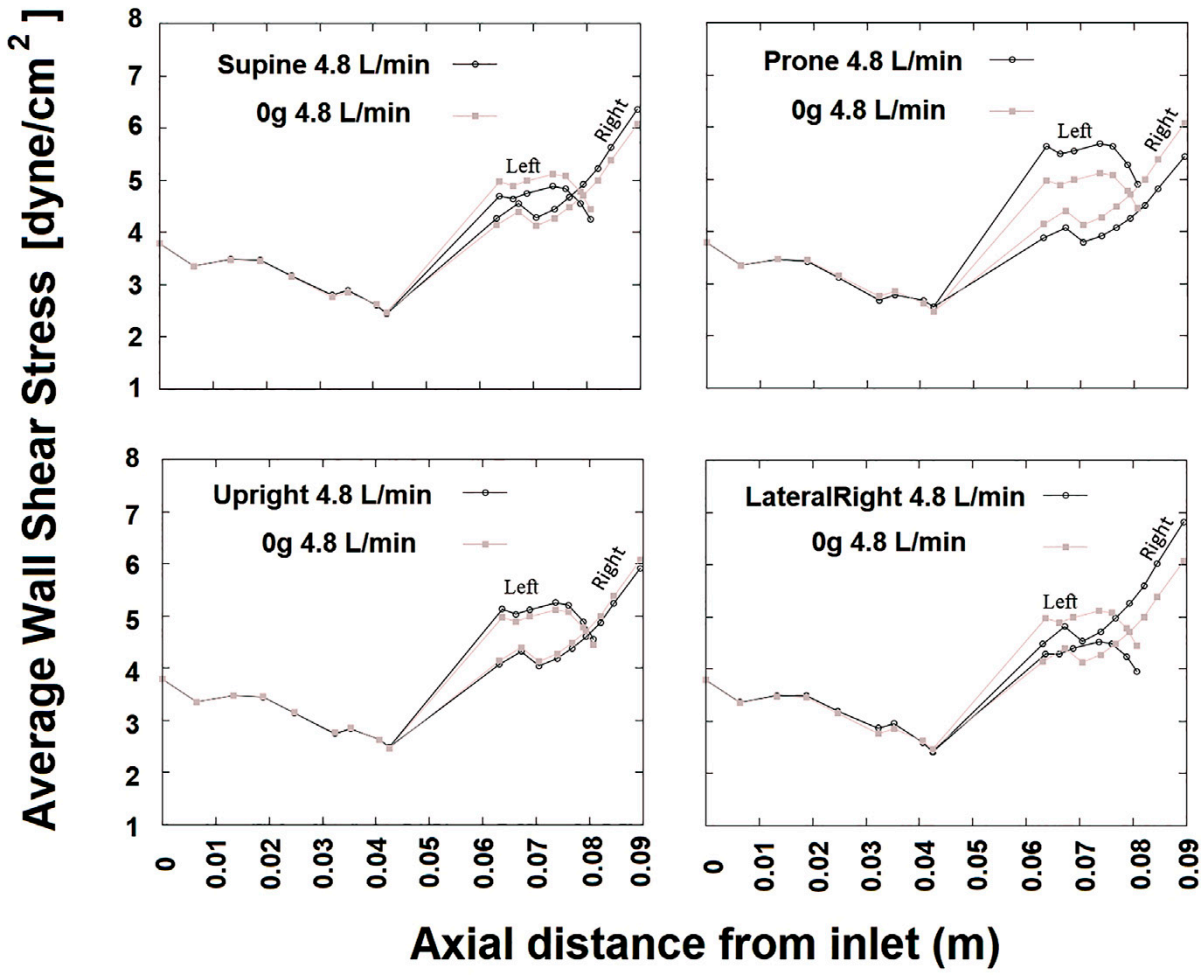
SAWSS (in Pascals) defined in axial planes plotted versus axial distance for cardiac output 4.8 L/min at different postures. Due to presence of the bifurcation, beyond an axial distance of approximately 0.04 m two curves are shown one for left and right. In each plot, results from 0g are shown for reference in gray. The RPA WSS in general is greater than that in the LPA.

Flow velocity magnitude and streamlines redistribute within the arterial lumen with posture (Figure 5). This also translates to a redistribution of shear stress with posture (Figure 6), with model predictions of shear stress differing in the LPA and RPA due to their relative size, and flow distribution shifts between the two lungs. Predicted RPA shear stress is typically higher than LPA shear stress, and assuming zero gravity simulations as a reference state (where flow is distributed relative to volume), both the left and right pulmonary arteries can exhibit shifts of up to 0.75 dyne/cm^2^ from this reference in between postures. The greatest shift simulated occurs between a prone and right lateral posture with a predicted difference of up to 1.35 dyne/cm^2^.

### Acinar Scale Perfusion Distribution

**FIGURE 7:**
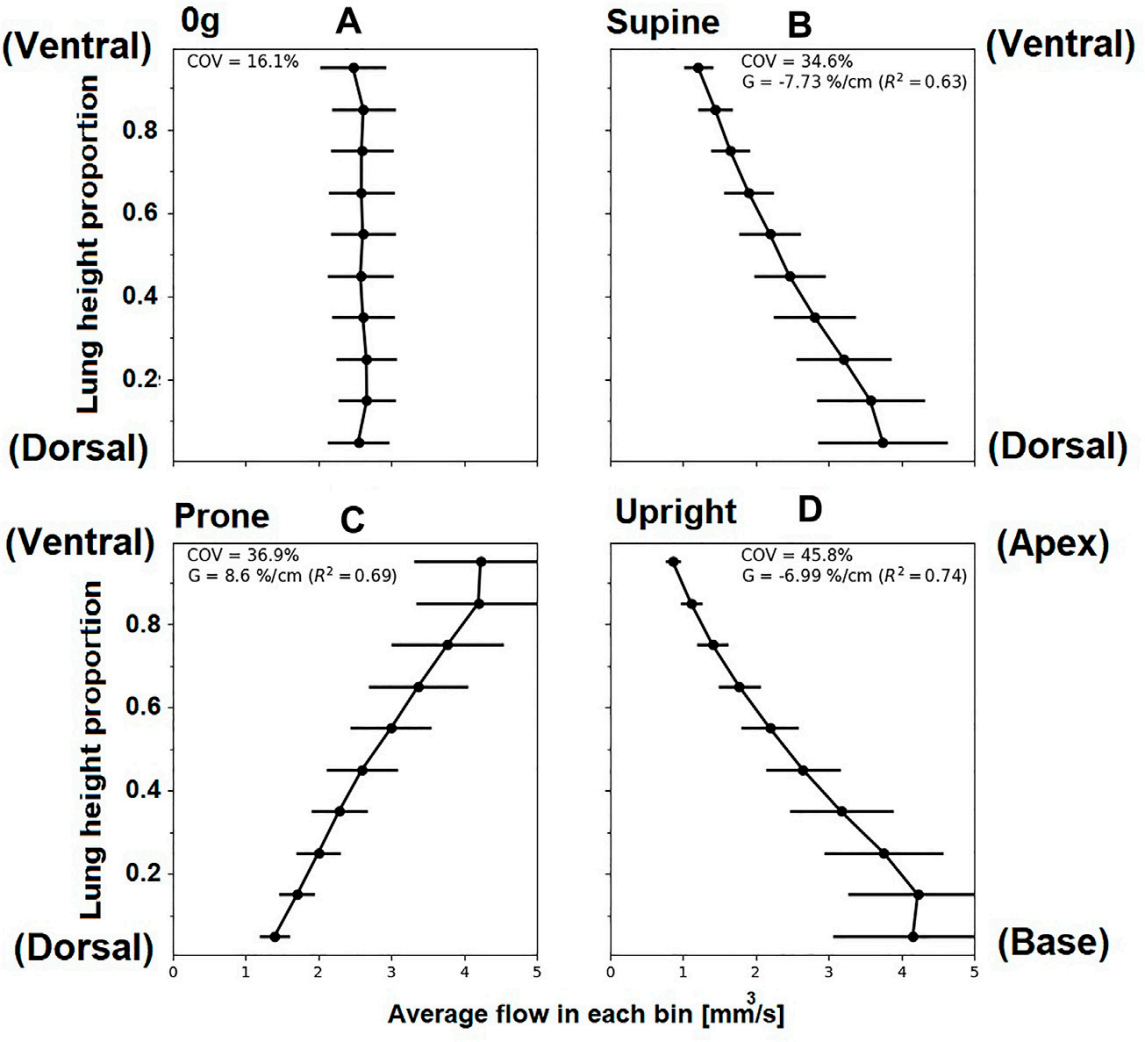
Simulated relationships between acinar perfusion and gravitational height for 4.8 *L*/min cardiac output in different postures. In each case, the coefficient of variation (COV) and the gravitational gradient (G) of perfusion is indicated. Panels show posture **(A)** zero gravity, **(B)** supine, **(C)** prone and **(D)** upright, subsequently. The y-axis shows proportional height in the gravitational direction (cranio-caudal in upright, and ventral-dorsal in supine/prone). For zero gravity (0g), no gravitational gradient is reported since without the presence of gravity this property cannot be defined, ventral dorsal height is plotted and other axes show similar results. Supine and prone show opposite slope direction as the gravity direction is the same but posture is inverted.

The coupled model predicts hemodynamics across spatial scales relevant to the lung. At the acinar scale, there is a known gravitational gradient in perfusion that is important in the matching of perfusion to ventilation. Figure 7 shows gravitational gradients in perfusion predicted by the coupled model in zero gravity, and under 1g in prone, supine (typically imaged) and upright (typical functional) postures at a fixed cardiac output. Comparisons with the same measures predicted by the 1D model and across different cardiac outputs are presented in Supplementary Section S2. Parameters for the right lateral posture are not reported as in this case the entire left lung is non-dependent tissue, and so gradients over the gravitational height become dependent on the lung in question. All predicted perfusion distributions are consistent with the previous model of Clark et al. (2011b), with gravitational gradients (G) in perfusion ranging from 6.80%/cm to 9.10%/cm and coefficient of variation (COV) ranging from 33.3%–47%. These ranges for G and COV have previously been shown to be consistent with imaging studies, when analysed on a spatial scale typical of imaging (e.g., the voxel size in magnetic resonance imaging) Clark et al. (2011b). The coupled model predicts physiologically consistent changes with cardiac output, that is a decrease in G as the gravitationally non-dependent lung vasculature is recruited, and a consistent decrease in COV as perfusion becomes more uniform over the height of the lung.

**FIGURE 8:**
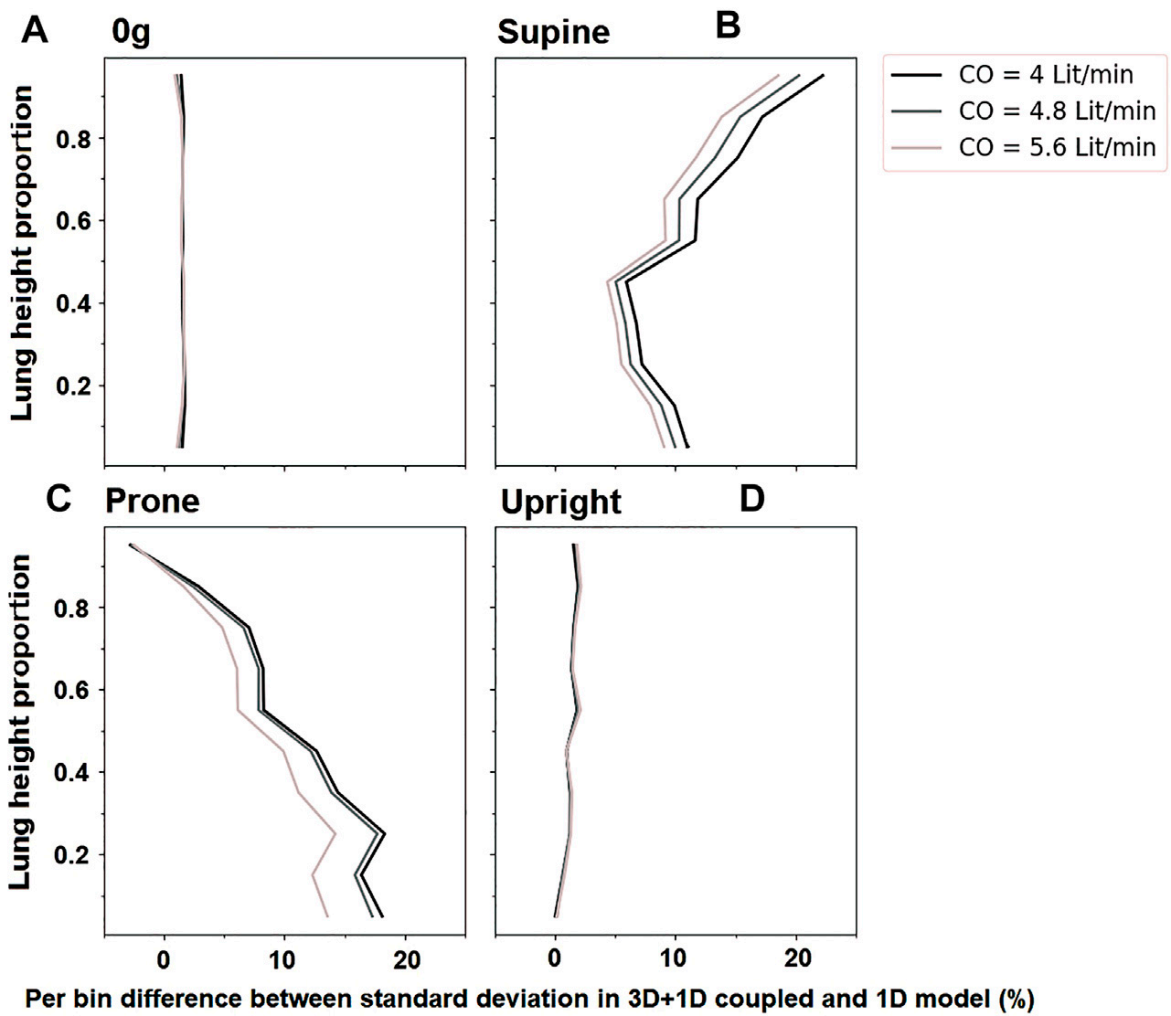
The change in standard deviation of acinar perfusion in simulations of the coupled 3D macro-scale model to a 1D micro-scale model compared to a full 1D simulation as previously published by Clark et al., 2011a. Simulated relationships are shown at different cardiac outputs (4 L/min, 4.8 L/min 5.6 L/min). The y-axis shows proportional height in the gravitational direction (cranio-caudal in upright, and ventral-dorsal in supine/prone). For zero gravity (0g), no gravitational gradient is reported. Standard deviation in different postures is shows as **(A)** For zero-gravity, **(B)** supine, **(C)** prone and **(D)** upright.

In general, the coupled model predicts a more heterogeneous distribution of perfusion within the lung than the 1D model previously presented by Clark et al. (2011b). This is due to the balance between resistive properties of the two scales of the model which results in a redistribution of blood flow between the two lungs in the 3D compared with the 1D model. Figure 8 illustrates the change in the predicted standard deviation of perfusion with gravitational height of the lung in the coupled model compared to the previously published 1D model Clark et al. (2011b). In zero gravity and upright simulations, the left and right lung flow distributions are approximately equivalent to the relative volume of the two lungs and so the two models predict a similar heterogeneity in perfusion with gravitational height. However, in the supine and prone lungs there is a redistribution of flow meaning that the left and right lungs do not receive a relative flow that matches their volume and so there is overall an increase in heterogeneity as predicted in the 3D model compared to the previously published 1D model. The effect is greater in the gravitationally non-dependent lung which accommodates increases in flow. As overall flow (cardiac output) increases to one lung or the other, the distribution of flow in that lung becomes more uniform, so the impacts of the coupling are most apparent at lower cardiac outputs. In these cases, an increase in acinar perfusion heterogeneity of up to 20% in the coupled model compared to the 1D model is observed, indicating the inter-connectedness between macro-scale and micro-scale function.

## Discussion

In this study, we have presented an open-source methodology to couple 3D macrovascular fluid mechanics simulations to an anatomically defined 1D network model of the distal vasculature to allow for multi-scale analysis of pulmonary hemodynamic function. The inclusion of an anatomic network model in this methodology provides unique opportunities to connect the complexity of macro-vascular fluid dynamics with a recruitable capillary bed West et al. (1964); West (1999), that sits within a stratified acinar structure Read (1969b) that responds to gravitational influences West et al. (1964); Hopkins et al. (2007).

Computational fluid dynamics of the pulmonary circulation has been used widely, particularly in the assessment of macro-vascular wall shear stress due to its role in the development of pulmonary hypertensive disease Bordones et al. (2018); Kheyfets et al. (2015); Kong et al. (2018); Tang et al. (2012). As 3D simulations do not typically cover the full vasculature, the choice of outflow boundary conditions is critical, and can significantly impact simulation accuracy Kheyfets et al. (2013). Common boundary conditions employed in models of the pulmonary circulation include zero traction, resistance, and Windkessel (resistance-compliance) which can be adjusted to account for subject-specific variability but do not capture the anatomy or function of the distal micro-vasculature Kheyfets et al. (2013). An alternative is the structured tree approach Kheyfets et al. (2015), which uses an analytical approximation for downstream resistance of each 3D model outlet to characterize the expected pulmonary vascular resistance. This is based on the area of the outlet vessel and expected change in vessel cross-sectional area with generation in a typical pulmonary vascular tree. These structured trees are typically based on the same morphometric data against which we have validated our anatomic 1D network model Horsfield (1978); Huang et al. (1996). However, they are not constrained by the shape of the lung nor informed by the function of the recruitable capillary beds in the lung. Zero gravity simulations in our model can be considered as similar to the structured tree approach as they estimate the downstream resistance based on extra-acinar vascular branching and the volume of the lung that is fed by a 3D model outlet.

While the distribution of perfusion in the lung has been simulated in whole lung models, these models typically employ simplified fluid dynamics, assuming arteries to be tubes within which flow is axisymmetric Clark et al. (2011b); Burrowes et al. (2005). While these simplifying assumptions allow for physiological predictions of perfusion within the lung which can be coupled to predictions of gas exchange (Burrowes et al. (2011b); Clark et al. (2014)), they do not hold in the largest pulmonary blood vessels where the flow is complex. In pulmonary hypertension, the flow patterns in the largest pulmonary arteries can exhibit vortices Schäfer et al. (2017). Disruptions to flow patterns due to bifurcations are also common in these larger arteries that have relatively high Reynolds numbers (see for example, Figure 5). These flow patterns introduce non-linearities between the flow rate in the major arteries and their resistance (flow dependent resistance, Figure 3). Flow dependent resistance is not typically captured in simplified network models of blood flow Clark et al. (2011b, 2014). Some models of airways have included a quasi-empirically derived resistance correction to account for this, but this correction is derived from experimentation and computational fluid dynamics simulations in representative geometries Pedley et al. (1970); Swan et al. (2012). Direct simulation in larger arteries is preferable to capture these effects in a patient specific manner. In addition to non-linearities in 3D models, 1D network models are typically also non-linear, due to their incorporation of vascular compliance and capillary recruitment, which occurs due to the balance of air and blood pressure at the acinar level. Capillaries can be locally collapsed, recruited and distended regionally in the lungs West et al. (1964); West (1999). This leads to a relatively large increase in flow with increases in pressure at low flows (as functional vascular bed is recruited), which then flattens as compliant limits are reached (consistent with whole lung pressure-flow relationships Burrowes et al. (2011a)). Although some simplified boundary conditions (e.g., Windkessel) can capture some of this non-linearity, the model presented here does so with an anatomical and physiological basis which can ultimately enable simulation of patient specific disruptions to resistance and compliance in conditions such as pulmonary hypertension.

Posture is an important consideration in assessing lung function, and it can have important implications in diagnosis and treatment of lung disease. For example, prone posturing has been shown to improve gas exchange in conditions such as respiratory distress syndrome Gattinoni et al. (2001), due to a redistribution of both ventilation and perfusion with gravity. In pulmonary hypertensive disease, some of the expected gravitational gradients in perfusion may be altered, as remodeling of small arteries and localized occlusion of vessels can lead to loss of vascular reserves and a more gravitationally uniform flow distribution, along with more locally heterogeneous blood flow Lau et al. (2014). The model presented in this study allows for gravitational influences to be investigated, and opens the door for studies that can investigate mechanisms of disruptions to these effects in disease. Here, we have shown that our coupled 3D-1D model can simulate changes in macro-vascular flow dynamics due to posture, simultaneously with predicting micro-vascular perfusion distribution. The volume of the two lungs are not equal, and the right lung has been reported to have 53.6 ± 1.5% of the total lung volume in supine (53.3 ± 1.3% upright) Yamada et al. (2020) reported. This is consistent with 0g simulations of flow distribution, albeit in a single patient-based model, which is distributed in a manner that is consistent with the differential in lung volume (53.4% to the right lung). The distribution changes with simulated posture, with the greatest flow to the right lung being in the lateral posture (58.8% of volumetric flow) and the lowest flow to the right lung being in the prone posture (50.5%) of volumetric flow. These re-distributions are consistent with imaging studies, which also show that with changes in posture, flow does not distribute proportionally to lung volume Wieslander et al. (2019), who showed on average 63% of flow distributing to the right lung in right lateral posture, compared with 52% in prone and 54% in supine. This redistribution of blood in our model is due to the shape of the lung, which means that in different postures there is a different volume of “dependent” tissue (tissue in which blood travels downward from the heart in the direction of gravity). Our previously employed 1D model does not exhibit the same physiological distribution of blood (see Supplementary Table S1), and this shows that at the macro-scale flow distribution is driven by both micro-vascular compliance-resistance relationships and the resistance of the upper vasculature. The left-right redistribution of blood with posture is also consistent with trends for higher heterogeneity in perfusion when using coupled model than a 1D model alone, which is consistent with observed perfusion heterogeneity Clark et al. (2011b). The dependence of blood flow distribution on posture does have functional implications for predictions of wall shear stress in computational fluid dynamics models, as imaged posture (usually supine) may not always reflect functional posture (often upright). We estimate that the effect of posture is likely to impact left and right pulmonary artery wall shear stress predictions by 0.75–1.35 dyne/cm^2^, which are comparable in magnitude to differences in time averaged wall shear stress predicted by computational fluid dynamics in patients at risk for or with mild pulmonary hypertension Pillalamarri et al. (2021). The redistribution of left-to-right blood flow with posture in the coupled model has implications for prediction of both flow velocity profiles and wall shear stress in computational fluid dynamics models of the major arteries. Most computational fluid dynamics models, including structured tree models, in essence assume a left-right flow distribution that relates to the relative size of model outlets. Zero gravity simulations reflect these conditions, however, the flow redistribution that our coupled model provides demonstrates that shifts in posture may be functionally important.

Assessment of pulmonary artery hemodynamics can be achieved using imaging techniques such as 4D-cine magnetic resonance imaging (CMRI) and functional magnetic resonance imaging (fMRI). The availability of such imaging to diagnose pulmonary hypertensive disorders can be an issue and also the cost of running such tests can be a burden to some patients. While these imaging modalities can help with understanding the state of disease in some cases, a better understanding of pulmonary artery hemodynamics obtained by computational fluid dynamics could lead to greater insight in conditions such as pulmonary hypertension and tetralogy of Fallot Hu et al. (2020); Schäfer et al. (2017, 2019). Imaging studies of the proximal pulmonary arteries have suggested a relationship between mechanical and flow hemodynamic domains Schäfer et al. (2017). Changes in WSS and viscous energy loss in MPA and RPA have also been observed in disease Hu et al. (2020); Schäfer et al. (2019), and models such as this could provide insight without the need to collect a significant amount of data that can be expensive and more time consuming. The ability of the presented model for patient-specific investigation could provide insights into hemodynamic assessment of both children and adults, where changes in the micro-structure of the lung may influence macro-vascular flow properties. This may occur differently in children compared with adults, with studies suggesting that flow hemodynamics goes through uniform changes in adults with pulmonary arterial hypertension whereas the flow abnormalities are more prevalent in children with pulmonary arterial hypertension Schäfer et al. (2019).

The coupled model presented here provides a strength in its prediction of both macro-vascular flow dynamics and micro-vascular perfusion. This provides a strong framework for future studies of the pathological lung, particularly in pulmonary hypertension. In an acute form of pulmonary hypertension (pulmonary embolism) network models have been used to predict the impact of vascular occlusion on pulmonary vascular resistance, and importantly on gas exchange function Burrowes et al. (2011a,b); Clark et al. (2014). These studies show that the location of a vascular occlusion (for example, does it occlude a region that typically receives a relatively high flow due to gravity?) impacts its functional importance, and that the location of occlusion has differential impacts on vascular resistance and exchange. Pulmonary embolism can lead to a chronic remodeling of the small pulmonary arteries, and ultimately chronic thromboembolic pulmonary hypertension (CTEPH), in which pulmonary vascular function becomes impaired and heterogeneously distributed Lau et al. (2014). Network models can simulate remodeling in CTEPH Colebank et al. (2021); Ebrahimi et al. (2019, 2021); Qureshi et al. (2014). However, without accurate coupling to macro-vascular models, measurable changes in flow dynamics and shear stress in the largest pulmonary arteries cannot be predicted. While we have used 3D computational fluid dynamics here to predict flow in the main, left and right pulmonary arteries, the methodology employed here is transferable to 3D simulation across scales of interest, for example to the level of segmental arteries. This could provide new insights into the progression of pathologies such as CTEPH in the future.

There are several coupling techniques that can be used to link the large scale (3D) effects and network (1D) flows within a single system. The coupling methodologies can be broadly divided into manual or automatic depending on the method of data transfer between models (one-way or two-way depending on whether both systems mutually influence each other or not). Passerini et al. (2009) proposed a method to simulate the blood flow in the circle of Willis in the brain, which assumed a rigid 3D domain and a compliant 1D model for their biological vessel domains. A Dirichlet-Neumann type mapping of vascular impedance was presented in Vignon-Clementel et al. (2006). A coupled 3D-1D model was first introduced by Formaggia et al. (1999) and followed up by Formaggia et al. (2001, 2002); Urquiza et al. (2006). A number of studies have taken coupling 3D compliant models with a reduced 1D model approach Formaggia et al. (1999, 2001); Urquiza et al. (2006). Their approach included maintaining a continuity of a hemodynamic quantity (flow rate or pressure) at the interface of two models. Blanco et al. (2007) introduced a novel method using variational formulation to minimize the error caused by dimension mismatch in the coupling. Our approach aims to reach an optimal compromise between computational cost and model accuracies across spatial scales.

To demonstrate the methodology we simulated 3D blood flow in the main pulmonary artery and the left and right pulmonary arteries. The number of generations modeled explicitly in CFD studies of the pulmonary arteries ranges from two Bordones et al. (2018); Capuano et al. (2019) to approximately 6 or 7 generations Kheyfets et al. (2015); Tang et al. (2011, 2012). The choice of upper artery CFD geometry depends on the application of the model, and influences simulation time. Although the methodology presented here is applicable to any number of generations in a 3D model, the primary aim of this study was to investigate the distribution of blood flow and wall shear stress predicted by a CFD model coupled to a 1D model that includes the effects of gravity and anatomy. The largest effects in flow distribution are expected to be at the left/right lung scale Wieslander et al. (2019), and flow dependent disruptions are expected to be most significant in the main, left and right pulmonary arteries Schäfer et al. (2017). Capuano et al. (2019) demonstrated that including additional bifurcations to a CFD model of the main pulmonary trunk did not significantly impact predicted flow distributions in the main, left, or right pulmonary arteries. Therefore, a two generation model was appropriate for this application. With this choice of geometry our model is able to be solved on a Desktop computer (Intel(R) Core(TM) i7-7700 CPU @ 3.60 GHz and 32GB RAM), with the 3D CFD model taking approximately 40 min to solve with parallelization on 4 cores, and the 1D model taking approximately 20 min to solve. These solve times can be reduced by using parallelization on high performance computers, and by choosing realistic initial conditions for simulations (for example, as the model approaches convergence, simulations from one iteration can initialize the next). Both 1D and 3D models must be solved at each coupling iteration, however, under normal parameterization this is well within normal solution times for CFD models (e.g., times cited by Tang et al. (2012)), with 25–30 iterations typically required for convergence.

Subject specificity is incorporated into the model via 1) a 3D description of the largest blood vessels, 2) a representation of branching architecture and vascular dimensions in vessels that can be resolved in CT (to one generation beyond the segmental level), and 3) in lung shape and volume. In participants with normal lung function patient-specific boundary conditions are difficult to derive as invasive clinical procedures are infrequent Kheyfets et al. (2015). However, in future studies of pulmonary hypertension inflow boundary conditions could be derived from clinical data including catheter measured flow and pressure profiles Tang et al. (2012), ultrasound Su et al. (2012), or magnetic resonance imaging Tang et al. (2011). An “atlas” based approach Capuano et al. (2019), may also be well-aligned with our modeling methodology. In this approach, statistically derived 3D models of the lungs and their vasculature could be applied with a range of typically measured boundary conditions in cohorts of patients, to understand variability in pulmonary function between groups, without the need to solve patient specific models for each participant in a large cohort.

The model assumes Newtonian behavior of the blood flow in the large arteries in both 1D and 3D models where the shear thinning of blood have no substantial impact, and hence blood viscosity is thought to be constant and irrespective of vessel radius. This assumption is applied in other studies, which have supported the assumption that the effects of non-Newtonian fluid on hemodynamics in the major pulmonary arteries are negligible Gao et al. (2013); Cho and Kensey (1991); Perktold et al. (1991). Non-Newtonian effects in the micro-vasculature are incorporated simply via an effective viscosity that depends on blood vessel size in the acinar structures Clark et al. (2011b). Our model also neglects vessel distensibility in the 3D geometry. In the steady state system that we simulate here, transmural pressure in the macro-vasculature is relatively consistent, and we do not consider the oscillations of blood pressure over a heart beat. Given that the CT imaging is acquired *in vivo* (at physiological pressures and volumes - 50% of vital capacity), this assumption of a rigid macro-vasculature is reasonable. However, more complex fluid structure-interaction models may improve accuracy in the future. A further improvement to the model would be the inclusion of transient changes in the vasculature. Passerini et al. (2009) developed a transient model which included a rigid macro-vasculature and a compliance-resistance model representing the distal blood vessels in the cerebral vasculature. The rigid vessel assumption could cause mismatch in coupling systems in the transient case, which could be addressed in a similar manner to assess WSS over a heart cycle, perhaps coupled to pulsatile corrections to our 1D steady state network model presented here Clark and Tawhai (2018). Previous CFD studies have suggested that the error in predicted spatially averaged WSS from static versus pulsatile simulations is small Kheyfets et al. (2015). However, pulsatile simulations in the future would provide important insights into dynamic changes in the circulation, which may play a role in response to disease Ebrahimi et al. (2021).

In this study, we introduce a novel 3D/1D coupled model of the pulmonary circulation that operates as a patient-specific model to investigate hemodynamics. An advantage of this model is having an anatomical 1D tree downstream of 3D which makes it provides a subject-based boundary condition to the 3D model. This model has the potential to be applied on a patient-specific manner to interrogate the effects of disease downstream on the upper vasculature. A further advantage of this model is its ability to simulate WSS in the upper vasculature in health and disease under different conditions such as posture and cardiac outputs. The model is designed to provide insights on the pulmonary vasculature to enhance the understanding of disease and help with clinical decision making.

## Data Availability

The datasets presented in this study can be found in online repositories. The names of the repository/repositories and accession number(s) can be found below: https://github.com/LungNoodle/lungsim.

## Footnotes

1
www.openfoam.org

2
https://github.com/LungNoodle/lungsim
