# Supplementary material for: Simulating Multi-Scale Pulmonary Vascular Function by Coupling Computational Fluid Dynamics With an Anatomic Network Model
Source: Front Netw Physiol. 2022 Apr 25;2:867551. doi: 10.3389/fnetp.2022.867551 (PMC10012968; doi:10.3389/fnetp.2022.867551)
Supplement: Supplementary file 1 [file DataSheet1.PDF]

Supplementary Material: Simulating multi-scale pulmonary vascular function by coupling computational fluid dynamics with an anatomic network model

1 TESTING COMPUTATIONAL FLUID DYNAMIC BEHAVIORS AGAINST AVAILABLE DATA

To test the computational fluid dynamics (CFD) simulations against available data, we chose to compare pulmonary artery velocity profiles from the literature. Bordones et al. (2018) simulated blood flow using CFD and generated experimental (3D printed phantom) comparisons. The velocity profiles for MPA and LPA were normalized by the inlet velocity and plotted against normalized diameter. Overall, the velocity profiles shows similarities in flow patterns. The alignment in results depicted in LPA velocity profiles is reasonable despite geometry differences between the two models as our model is not a replica of Bordones et al. Bordones et al. (2018).

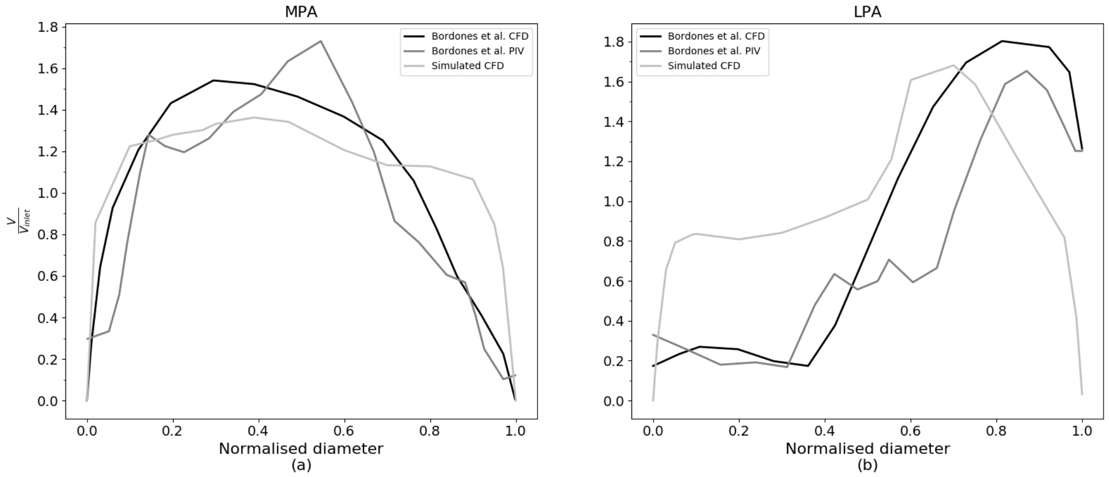

Figure S1. Velocity profile comparison with experimental (Phantom) and CFD results in described previously by Bordones et al. (2018)

Figure S2 depicts the WSS distribution contours over the main pulmonary artery surface in different postures. The distribution follows similar patterns, however, there are regional differences in RPA and LPA between postures. The inlet of the geometry has relatively high WSS due to uniform velocity profile at the inlet, leading to a transition region. The assumption of parabolic velocity profile (fully developed flow) was not used as the inlet boundary condition due to studies that suggest a uniform velocity profile in the pulmonary artery Miyasaka et al. (1993); Reuben et al. (1970); Tang et al. (2001). The results show that LPA has higher regional WSS in prone while RPA has shows some higher regional WSS in supine compared to other postures. The areas where flow separates from the wall low shear stress is observed.

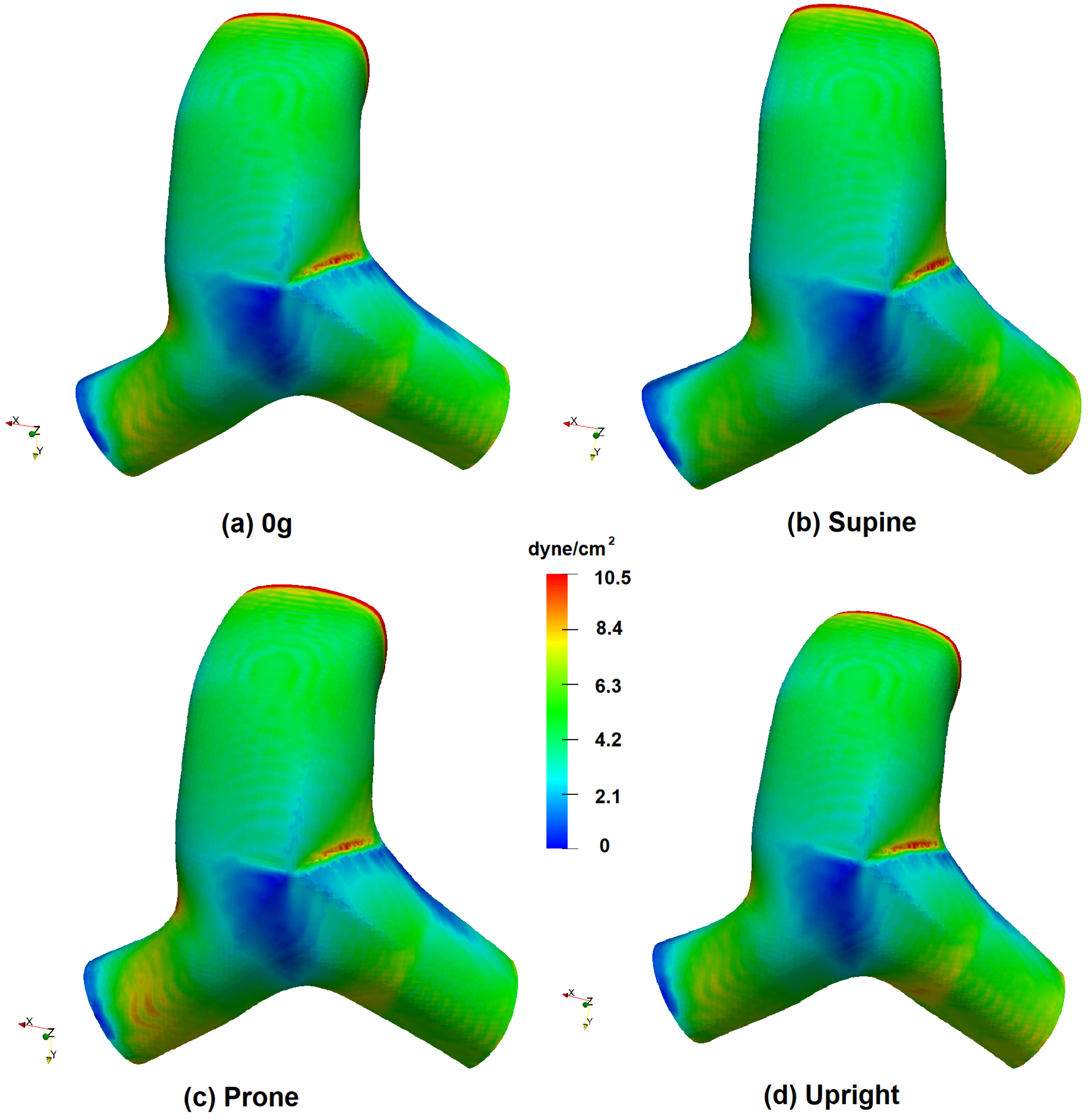

Figure S2. Comparison of WSS relative to postures changes with WSS contours in 4.8 l/min CO for different postures for the 3D part of the coupled model. Differences in WSS distribution close to the outlets are observed in each case.

2 MODEL BEHAVIORS ACROSS DIFFERENT CARDIAC OUTPUTS AND POSTURES

Table S1 shows the calculated flow entering each pulmonary artery branch across different postures and cardiac outputs. Results are shown for both the coupled model, and for a complete 1D network model (with no detailed macro-vascular simulation). Overall, a greater proportion of flow enters the right pulmonary artery (RPA) and into the right lung, which is typically the larger lung. The results suggest that in absence

of the gravity and upright postures the 1D model and coupled model show good agreement, although in the supine and prone postures there is a larger discrepancy between the models, and model predictions move closer to expected flow distributions when the model is coupled.

Table S1. Flow percent split between right and left pulmonary arteries in both 1D and coupled model with respect to changes in cardiac output (CO) and postures.

		0g		Supine		Prone		Upright	
		LPA	RPA	LPA	RPA	LPA	RPA	LPA	RPA
CO = 4 l/min	1D	46.5%	53.5%	46.5%	53.5%	46.2%	53.8%	46.8%	53.2%
	Coupled	46.5%	53.5%	43.1%	56.9%	48.7%	51.3%	46.5%	53.5%
CO = 4.8 l/min	1D	46.5%	53.5%	46.5%	53.5%	46.2%	53.8%	46.8%	53.2%
	Coupled	46.6%	53.4%	43.3%	56.7%	49.5%	50.5%	46.8%	53.2%
CO = 5.6 l/min	1D	46.5%	53.5%	46.5%	53.5%	46.2%	53.8%	46.8%	53.2%
	Coupled	46.7%	53.3%	43.5%	56.5%	49.1%	50.9%	46.5%	53.5%

Figure S3 shows the acinar blood distribution across both lungs with different postures and cardiac outputs. The average flow was calculated at the acinar level across ten different bins alongside the gravitationally dependent direction for every posture. In general, the average distribution at acinar level shows agreement between 1D and coupled models while the standard deviation distribution is typically larger in the coupled model (see Figure 8 in the main manuscript). Table S2 shows perfusion gradients in the supine, prone and upright postures and different cardiac outputs per acinus. There is general consistency between coupled and 1D models, and an expected reduction in the magnitude of perfusion gradients with increasing cardiac output. Table S3 summarizes the coefficient of variation (COV%) in acinar flow in both 1D and coupled models for each posture and cardiac outputs. The least COV% is observed zero gravity, which is expected as a result of a lack of gravitational gradients. The coupled model typically shows a higher COV% than the 1D model due to redistribution of blood between left and right lungs. However, the impact on overall heterogeneity is small.

Table S2. Perfusion gradient comparison in 1D and coupled model of acinar units in gravitational direction for different postures and different cardiac outputs. Cardiac outputs (CO) simulated are at 4, 4.8 and 5.6 l/min. G is the gravitational gradient which is the blood flow gradient considering each acinus regressed against gravitational height.

		CO = 4 l/min		CO = 4.8 l/min		CO = 5.6 l/min	
		1D	Coupled	1D	Coupled	1D	Coupled
Supine	G, %/cm	-7.94	-7.94	-7.73	-7.73	-7.53	-7.53
	R^2	0.68	0.64	0.66	0.63	0.64	0.61
Prone	G, %/cm	9.11	8.95	8.78	8.61	8.44	8.33
	R^2	0.77	0.72	0.75	0.69	0.73	0.71
Upright	G, %/cm	-7.25	-7.22	-7.03	-6.99	-6.80	-6.77
	R^2	0.77	0.76	0.75	0.74	0.74	0.73

REFERENCES

Bordones, A. D., Leroux, M., Kheyfets, V. O., Wu, Y.-A., Chen, C.-Y., and Finol, E. A. (2018). Computational fluid dynamics modeling of the human pulmonary arteries with experimental validation. *Annals of Biomedical Engineering* 46, 1309–1324

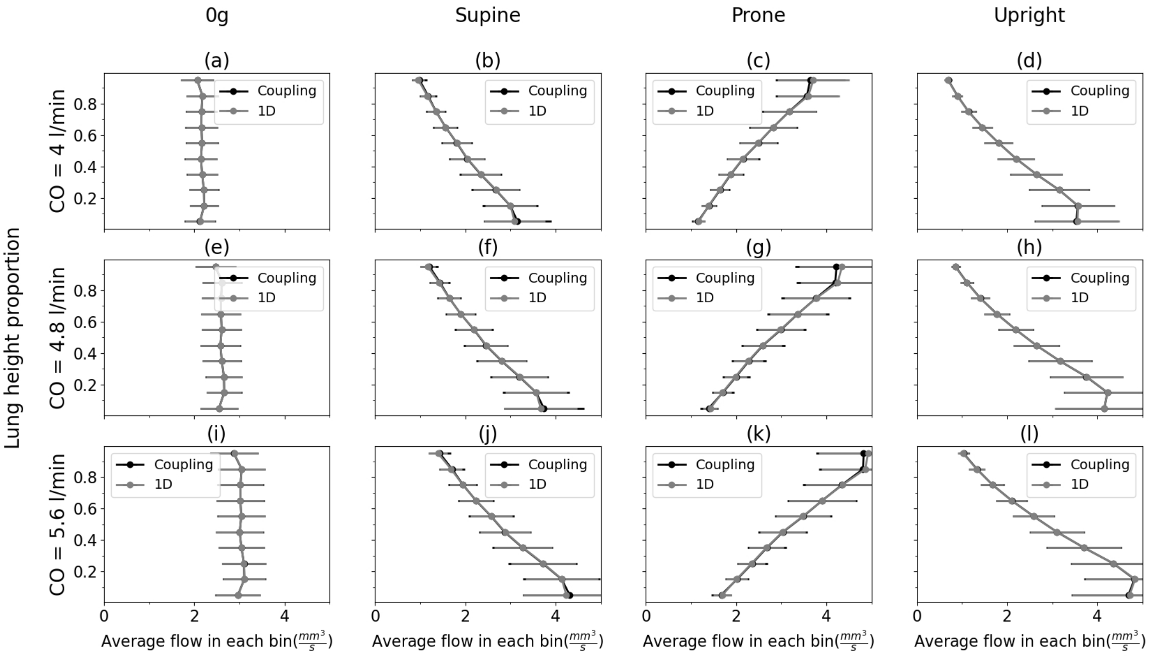

Figure S3. Flow distribution at the acinar level for different postures (columns) and cardiac outputs (rows) against lung height proportion in gravitational direction in both 1D and coupled models.

Table S3. Comparison of model predictions of COV% of the acinar units flow in purely 1D model with coupled in different postures and different cardiac outputs.

	CO = 4 l/min		CO = 4.8 l/min		CO = 5.6 l/min	
	1D	Coupled	1D	Coupled	1D	Coupled
0g	15.8%	16.1%	16.3%	16.5%	16.7%	16.9%
Supine	34.1%	35.1%	33.7%	34.6%	33.3%	34.1%
Prone	36.7%	36.9%	35.1%	36.1%	34.8%	35.2%
Upright	46.8%	46.8%	45.8%	45.8%	44.7%	44.7%

Miyasaka, K., Takata, M., and Miyasaka, K. (1993). Flow velocity profile of the pulmonary artery measured by the continuous cardiac output monitoring catheter. *Canadian journal of anaesthesia* 40, 183–187

Reuben, S., Swadling, J., and Lee, G. d. J. (1970). Velocity profiles in the main pulmonary artery of dogs and man, measured with a thin-film resistance anemometer. *Circulation research* 27, 995–1001

Tang, T., Chiu, S., Chen, H.-C., Cheng, K.-Y., and Chen, S.-J. (2001). Comparison of pulmonary arterial flow phenomena in spiral and lecompte models by computational fluid dynamics. *The Journal of thoracic and cardiovascular surgery* 122, 529–534
